# Advancements in Pancreatic Cancer Detection: Integrating Biomarkers, Imaging Technologies, and Machine Learning for Early Diagnosis

**DOI:** 10.7759/cureus.56583

**Published:** 2024-03-20

**Authors:** Hisham Daher, Sneha A Punchayil, Amro Ahmed Elbeltagi Ismail, Reuben Ryan Fernandes, Joel Jacob, Mohab H Algazzar, Mohammad Mansour

**Affiliations:** 1 Internal Medicine, University of Debrecen, Debrecen, HUN; 2 Internal Medicine, University Hospital of North Tees, Stockton-on-Tees, GBR; 3 Neurology, University of Debrecen, Debrecen, HUN; 4 Vascular Surgery, The Royal London Hospital, London, GBR; 5 General Medicine, Diana Princess of Wales Hospital, Grimsby, GBR; 6 General Surgery, Karcagi Katai Gabor Korhaz, Karcag, HUN; 7 General Medicine, University of Debrecen, Debrecen, HUN; 8 General Medicine, Jordan University Hospital, Amman, JOR

**Keywords:** pancreas disease, ethics in ai, ai and machine learning, ai cancer detection, ai in cancer treatment, pancreatic cancer detection, pancreatic cancer diagnosis, ai & robotics in healthcare

## Abstract

Artificial intelligence (AI) has come to play a pivotal role in revolutionizing medical practices, particularly in the field of pancreatic cancer detection and management. As a leading cause of cancer-related deaths, pancreatic cancer warrants innovative approaches due to its typically advanced stage at diagnosis and dismal survival rates. Present detection methods, constrained by limitations in accuracy and efficiency, underscore the necessity for novel solutions. AI-driven methodologies present promising avenues for enhancing early detection and prognosis forecasting. Through the analysis of imaging data, biomarker profiles, and clinical information, AI algorithms excel in discerning subtle abnormalities indicative of pancreatic cancer with remarkable precision. Moreover, machine learning (ML) algorithms facilitate the amalgamation of diverse data sources to optimize patient care. However, despite its huge potential, the implementation of AI in pancreatic cancer detection faces various challenges. Issues such as the scarcity of comprehensive datasets, biases in algorithm development, and concerns regarding data privacy and security necessitate thorough scrutiny. While AI offers immense promise in transforming pancreatic cancer detection and management, ongoing research and collaborative efforts are indispensable in overcoming technical hurdles and ethical dilemmas. This review delves into the evolution of AI, its application in pancreatic cancer detection, and the challenges and ethical considerations inherent in its integration.

## Introduction and background

Since the term artificial intelligence (AI) was coined in the 1950s, it has continued to evolve with the emergence of tools such as machine learning (ML) and deep learning (DL) [1]. In the field of medicine, AI has been applied to aid in cancer diagnosis and treatment. The use of its algorithms in onco-imaging and onco-pathology for cancer screening, tumor grading and staging, and the overall clinical decision-making process is gradually becoming invaluable in caring for cancer patients [2]. 

While the predictive capabilities of AI are significant and valuable, it is imperative to address issues like standardization, validity, ethics, privacy, and financial considerations before fully leveraging its potential in diagnostic oncology [3]. Pancreatic cancer, which accounts for 4% of all cancer-related deaths, ranks as the seventh leading cause of cancer deaths worldwide. Being a significant contributor to global cancer mortality, the need for innovative approaches to enable its early detection and intervention is paramount [4-6]. Clinically, pancreatic cancer, predominantly pancreatic ductal adenocarcinoma, arises from abnormal DNA mutations in the pancreatic ductal cells [7,8].

While surgical resection remains the primary curative treatment method, the late-stage presentation limits the number of eligible patients. When surgical resection is feasible, usually through a pancreaticoduodenectomy procedure, the survival and recurrence rates remain poor, even after complete resection [9]. In light of these challenges and the limited effectiveness of current diagnostic methods, this review endeavors to examine the pivotal role of AI in facilitating early detection of pancreatic cancer, and thereby ultimately improving patient outcomes.

## Review

Current and emerging methods of pancreatic cancer detection

Historically, individuals with pancreatic cancer have had a poor prognosis since they are often diagnosed at advanced stages of the disease. Long-term survivors are often those who present with incidental imaging findings while still at stage I or II, enabling surgical resection to be curative [10]. Histopathological analysis and cytological specimens are commonly used today to detect pancreatic cancer, and these samples are commonly obtained using CT-guided biopsy, endoscopic ultrasonography (EUS), exploratory biopsy using either laparoscopic or open surgery, and ascites cytology. Tumor markers are also used to look for potential signs of pancreatic cancer, especially in high-risk individuals such as those with a predisposition based on genetic markers or a history of chronic pancreatitis. The six tumor markers that are on the watch list in such cases are carbohydrate antigen 19-9 (CA19-9), carcinoembryonic antigen (CEA), CA242, microRNAs, CA125, and K-RAS gene mutations [11]. Other markers that aid in the detection include TP53, circulating cell-free DNA (CfDNA), mutation-specific CfDNA, Smad4, PDAC1, and CDKN2A [12,13]. CA19-9 remains by far the most commonly used to predict prognosis and monitor recurrence, especially postoperatively [14,15].

Clinical findings that raise suspicion for a malignant pancreatic lesion, requiring comprehensive cancer screening, include cysts greater than or equal to 3 cm, thick cyst walls, increased CA19-9, and a fast rate of growth of greater than 5 mm over two years. In such scenarios, the chances of invasive cancers are higher, often necessitating surgical resection. Molecular imaging and pancreatic juice analysis still require further research, potentially opening up possibilities of using AI to aid in pancreatic cancer detection [16]. Currently, screening tests are only advised for individuals with high-risk factors including familial predisposition, genetic factors such as Lesch-Nyhan syndrome, and chronic pancreatitis. A study published by Klatte et al. in 2022 analyzed data accumulated over 20 years using MRI with or without EUS. The study managed to detect 36 pancreatic ductal adenocarcinomas (PDAC) in 347 carriers of a germline CDKN2A mutation with a cumulative incidence of 20.7% by the 70-year age mark. Resectable cases accounted for 83.3% of them and they had a 32.4% five-year survival rate, highlighting the significance of optimized cancer detection in improving patient outcomes [17].

Screening programs for the detection of pancreatic cancer have been attempted in the past. However, due to its low incidence in the general population, where the lifetime risk is less than 2%, even a highly specific test can lead to many false positives, causing subjects to undergo surgical procedures and unnecessary medical, mental, and financial strain [18]. This strain can be reduced by prioritizing high-risk groups, including patients with familial or inherited risk, such as germline mutations in certain genes and a family history of PDAC [19,20]. Family history indicates a 50% lifetime risk in these individuals [21]. Cancer syndromes including Lynch syndrome 2, hereditary breast-ovarian cancer syndrome (HBOC), familial atypical multiple mole melanoma (FAMM), Li-Fraumeni syndrome, and Peutz-Jeghers syndrome, also increase the risk of PDAC. The mode of inheritance for these associated syndromes is mostly autosomal dominant [22]. New-onset diabetes mellitus (DM) is also a cause of concern. Patients with long-standing DM of more than five years are reported to have a moderately increased risk of 1-1.5-fold but those with new-onset DM (<1 year) have been reported to have a substantially increased risk for developing PDAC [23]. 

The current international guidelines state that screening high-risk individuals based on risk factors is the standard operating procedure to follow. Screening is recommended in patients whose lifetime risk exceeds 5%. This includes individuals with two first-degree relatives with PDAC, genetic syndromes (germline mutations in BRCA2, CDKN2A, or PALB2, or Li-Fraumeni syndrome or Peutz-Jeghers syndrome), and mucinous cystic lesions [24]. Adding to the above-mentioned factors are the modifiable risk factors such as smoking and obesity and the non-modifiable risk factors such as age, new-onset diabetes, and chronic pancreatitis. All of these factors must be considered for risk stratification [25].

Several diagnostic methods, with varying precision, have been employed for screening purposes. For instance, transabdominal ultrasonography (TUS), although considered safe and efficient from a radiation standpoint, has below-par diagnostic performance in this regard [26]. CT on the other hand has a good track record of 90% in finding solid pancreatic lesions and nodules. However, small tumors of <2 cm can be easily missed due to their iso-attenuating potential [27]. According to a study published in 2020, AI software has been used extensively in thoracic imaging to detect changes in histogram characteristics, texture, and shape parameter differences due to their ability to aid the human eye in identifying pathological differences [28-30]. The use of ML and algorithms of special neural networks with multiple levels for detecting features from the input data is a promising venture that can be of great use in healthcare and cancer detection in particular [31,32].

Pancreatic cancer biomarkers

Identification and Validation of Biomarkers

Biomarkers are measurable biological molecules that can be found in blood, tissues, and other bodily fluids. They can be employed as indicators of pathogenic processes, where they aid in early-stage pancreatic cancer screening, differential diagnosis, and distinguishing benign from malignant findings. They can also help with predicting the tumor's behavior, often guiding treatment options and evaluating response to therapy. Following the identification of possible biomarkers, a series of analyses are conducted to confirm the utility of a novel biomarker. Once an assay is validated, the biomarker is assessed further for additional evidence to verify that it is clinically valid and can be used in clinical practice [33,34].

A meta-analysis by Hagen et al., which was published in 2018, contributed to the identification and validation of a multi-gene biomarker panel for PDAC prognosis and diagnosis. Transcriptomes of 18 fresh-frozen tissues with a tumor content of 15-80% and 13 non-tumor pancreatic tissues were analyzed using Illumina humanRef-12 BeadArray. Numerous elevated pathways, such as T-cell antigen receptor (TCR), tumor necrosis factor-alpha (TNFα), transforming growth factor beta receptor (TGFβR), mitogen-activated protein kinase (MAPK), and integrin signaling, were successfully identified and classified into four clusters (1-4). Cluster 1 genes were downregulated in tumor tissue compared to non-tumor tissue, while clusters 2-4 genes were upregulated in tumor tissue. Gene expression data from 178 individuals with pancreatic cancer were examined in an attempt to establish a correlation between the genes and patient survival. This analysis found a correlation between clusters 2-4 and survival, indicating that they indeed have prognostic importance to pancreatic cancer patients and thereby underscoring the utility of biomarkers in pancreatic cancer detection and management [35].

Challenges in Identifying Reliable Biomarkers

Finding reliable biomarkers poses several difficulties, particularly when it comes to pancreatic cancer. These obstacles are exacerbated by the complexity of the disease and the shortcomings of the available diagnostic techniques [36,37]. Patients with pancreatic cancer vary greatly in their molecular features and genetic alterations, and this heterogeneity could make it difficult to identify distinct biomarkers for different disease stages or subtypes because a single biomarker may not adequately capture these variations [36]. Biomarkers are not always exclusive to a particular disease. This lack of specificity makes it difficult to identify and use them, especially in diseases like pancreatic cancer. For instance, the commonly used biomarker CA19-9 for pancreatic cancer diagnosis is not thought to be a suitable marker for the mass screening of early-stage pancreatic cancer. This is because it can be raised in other kinds of gastrointestinal adenocarcinomas and several benign disorders. Moreover, 5-10% of people lack the enzymes necessary for the production of CA19-9 and, thus, are ultimately unable to express it [37].

The limitations of ELISA assay detection or the signal created by imaging techniques that utilize metabolic pathways (18F-FDG-PET) or employ affinity reagents like tagged antibodies have restricted the measurement of protein biomarkers. High-quality affinity reagents, such as antibodies, are essential to both imaging and nanodetection methods. Unfortunately, the shortage of highly specific monoclonal antibodies renders the clinical translation of many prospective biomarkers complicated [38]. Due to the above-mentioned reasons, the identification and utilization of reliable biomarkers for cancer screening and detection continue to be challenging.

Recent Developments in Biomarker Research

Intending to improve the efficiency of cancer diagnosis and treatments, there are constant new advancements in the field of biomarker research to help in early cancer detection. For instance, extracellular vesicles (EVs) have a critical role as practical cancer biomarkers, according to mounting data. EVs, which are tiny, membrane-bound particles that have been isolated from bodily fluids like blood, saliva, and amniotic fluid, are becoming more and more popular as cancer biomarkers. Moreover, next-generation sequencing (NGS) technologies enable the simultaneous investigation of a wide range of genomic abnormalities, such as mutations, copy number variations, and the fusion of several genes. Hence, they have gradually replaced traditional techniques like FISH and qRT-PCR. Consequently, compared to serial single biomarker analysis, it is a more effective, economical, and tissue-saving tumor analysis method. Furthermore, biosensors are among the most effective clinical biomarker detection instruments for cancer diagnosis and prognosis. According to recent research published in 2022, electrochemical biosensors based on nanomaterials may offer a quick, sensitive, and practical way to identify cancer biomarkers [39].

The possibility for biomarker identification has also increased due to technological advancements, particularly with the creation of massive biological multi-omics datasets and AI algorithms [40]. Using ML and large-scale transcriptomic profiling of data from 1,665 non-tumorous tissue samples and 2,316 HCC, Kaur et al. identified three platform-independent diagnostic genes - FCN3 (downregulated in HCC), CLEC1B (downregulated in HCC), and PRC1 (upregulated in HCC) - that demonstrated prognostic potential and could detect HCC with high precision (93-98%) in both training and validation datasets, demonstrating the contribution of AI-powered tools to the precise identification of relevant biomarkers [41]. To create a screening algorithm that distinguished between normal and HCC tissue, Gholizadeh et al. also investigated mRNAs using ML. Their paper, which was published in 2023, revealed four additional prognostic markers (SOCS2, MAGEA6, RDH16, and RTN3) in addition to three diagnostic biomarkers (CYP2E1, ARK1C3, and AFP) [42]. In 2021, a DL tool was devised by Liang et al. to predict the risk of HCC diagnosis within a year by combining information from imaging, histology, electronic health records, and molecular biomarkers into a convolutional neural network (CNN); 9,553 out of 47,945 participants in this study had HCC, and the area under the curve (AUC) for predicting HCC risk one year ahead of time was 0.94, highlighting the collective power of AI in pooling clinical and diagnostic findings to predict the presence of cancer [43].

For predicting the effectiveness and response to treatment for tumors, biomarkers are crucial, and, in recent years, AI has begun to facilitate this role. In 2022, Hsu et al. evaluated how blood biomarkers, such as AFP, albumin-bilirubin (ALBI) grade, and circulating angiogenic factors, predict the efficacy of lenvatinib in patients with unresectable HCC using a decision tree-based survival predicting model that uses a random forest algorithm [44]. AI-based biomarker research in cancer has enormous promise to improve patient outcomes and revolutionize care, despite the challenges. Sustained investigation and cooperation are therefore necessary to guarantee that its potential is fulfilled in clinical application [29-44].

AI-Driven Biomarker Analysis

The ability of AI to analyze biomarker data is outstanding. With the use of relevant algorithms, AI can analyze huge databases to detect patterns and correlations within biomarker data. It can help detect relationships between cancer characteristics and biomarkers that may not be detectable by human analysis. AI analysis of patient data including genomics, transcriptomics, proteomics, and clinical information can assist in detecting biomarkers associated with pancreatic cancer that can help in setting treatment plans and prognoses [35,45].

Combining multiple biomarkers and analyzing them by AI can aid in the development of highly accurate models and algorithms that are capable of detecting pancreatic cancer in its early stages, staging, prognosis prediction, and therapeutic response prediction [35]. For instance, in 2020, Yang et al. created a multi-analyte panel that included miRNAs and mRNAs, cfDNA, and CA19-9. These data were utilized to train various algorithms. When used to diagnose PDAC, the model had an accuracy of 92%. For disease staging, the model achieved an accuracy of 84% [46].

In 2021, Li et al. gathered biomarker information on pancreatic cancer patients from various institutions and utilized six ML algorithms to build predictive models. Accurate predictions of one-year and two-year recurrence were made with the support vector machine (SVM) and K nearest neighbor (KNN) algorithm models, at 70.9% and 73.4%, respectively. Therefore, AI has the potential to be a valuable tool in detecting relapse in pancreatic cancer patients [47].

Traditional and AI-enhanced imaging methods

Traditional Imaging Methods in Pancreatic Cancer

CT, ultrasound, EUS, MRI, and PET are examples of imaging techniques often used for the investigation of pancreatic cancer. Multidetector CT (MDCT) has been developed as a cornerstone in diagnosis and staging in many countries, and EUS is clinically complemented by it, allowing access to fine needle aspiration (FNA). When CT and EUS are not diagnostic, magnetic resonance cholangiopancreatography (MRCP) and PET scanning can also play a successful role as supplementary imaging modalities [48,49].

On CT, pancreatic cancer is seen with abundant fibrous stroma and hypo-vascularity. Hence, it will subsequently appear hypo-attenuated or rarely iso-attenuated on contrast enhancement [50,51]. Abdominal US will show a hypoechoic mass possibly in conjunction with dilated pancreatic and common bile ducts [52]. EUS is superior to CT, MRI, and PET for the detection of small tumors and lymph node metastases [53]. Performing an MRI or enhanced MRI will show a hypointense lesion on T1-weighted and an iso/hyperintense lesion on T2-weighted imaging. The added contrast provides better accuracy with vascular involvement and local extension of the tumor [54,55]. Lastly, PET is clinically useful for its prognostic value and for monitoring treatment [56].

Limitations of Conventional Imaging

There is still an overlap between the imaging results of several pancreatic and peripancreatic disease processes, despite advancements in multimodal imaging. On ultrasonography, CT, and MRI scans, non-neoplastic pancreatic and peripancreatic entities may resemble primary pancreatic neoplasms. On the other hand, primary pancreatic cancer could go unnoticed during imaging [57]. Nonetheless, by distinguishing them based on their typical clinical presentation and imaging features, invasive diagnostic procedures or surgery may be avoided [58].

AI-Enhanced Imaging Technologies

Present-day AI has several promising applications in medical imaging. Firstly, virtual native enhancement (VNE), a cutting-edge technique, creates images that resemble virtual late gadolinium enhancement (LGE) without requiring contrast, thereby eliminating contrast-related concerns [59]. Secondly, an additional AI-aided reader helps in the early detection of breast cancer, where an AI assistant performs scans and highlights areas of interest for re-evaluation, improving diagnostic accuracy [60]. Similarly, a deep learning automated detection (DLAD) algorithm is designed to distinguish between normal and abnormal chest radiographs, and further identify several forms of major thoracic disease. This algorithm has consistently outperformed clinicians’ and thoracic radiologists’ diagnostic abilities, demonstrating its potential to enhance the quality and efficiency of clinical diagnoses [61].

Moreover, there has been significant development in AI applications in imaging and diagnosis of pancreatic cancer. In 2019, CNN, a type of artificial neural network (ANN) used for image recognition, was trained using a database of 4,385 CT images and later subjected to the analysis of CT scans from 100 patients, pre-assessed by three imaging specialists. The CNN demonstrated high accuracy and a faster diagnostic capability; CNN required only three seconds to arrive at a diagnostic finding, compared to eight minutes by an imaging specialist [62]. In the same year, Muhammad et al. used ANNs with a sensitivity and specificity of 80.7% to effectively predict and stratify the risk of pancreatic cancer as low, medium, or high risk based on personal health data. Their work demonstrates the potential of AI-based prediction systems for efficient risk management of pancreatic cancer, even in the absence of symptoms [63].

In a fascinating study published in 2021, a DL model-based computer-assisted diagnosis (CAD) system was created to evaluate EUS images and detect pancreatic cancer, chronic pancreatitis (CP), and normal pancreas (NP). There were 920 EUS photos in the training set and 470 EUS images in the testing set. In the testing and validation stages, the detection efficiency was 94% and 92%, respectively [64].

Machine learning algorithms for early detection

Overview of Machine Learning in Healthcare

ML is a subsection of AI that enables computers to compute and ingest large sums of raw data by utilizing algorithms that run input data. Through this process, it can predict the output values within a specific range of accuracy, simultaneously identifying different trends and varying patterns. The early development of ML in healthcare dates back to the 1950s and 1960s, coinciding with the arrival of computers and their potential applications in medical diagnosis and decision-making. During this period, researchers began to explore computational approaches to assist healthcare professionals in analyzing medical data and making informed decisions. Notably, this era witnessed the emergence of rule-based systems that operated on predefined rules and knowledge bases, enabling them to perform tasks such as diagnosis and recommendation within specific domains of expertise. These early endeavors laid the groundwork for subsequent advancements in ML and its integration into various aspects of healthcare delivery [65]. ML has continued to grow exponentially in recent years due to its semi-automated functionality that can fuse many different aspects of complex medicine and research data into one database, culminating in an output that might potentially have higher specificity and sensitivity [66,67].

Types of Machine Learning Algorithms

ML includes many different computer algorithms with each algorithm having particular advantages and disadvantages. The major types of algorithms are broadly classified as supervised and unsupervised learning. Supervised learning is a form of ML that requires entering both the input and output data into the model dataset, the main goal of which is to learn new functions or predictions on new unseen data. There are two main subsets of supervised learning: classification and regression. In classification, the algorithm is meant to assign the new input data to a specific category, e.g. diagnosing CT images as liver cancer or gallbladder cancer. Meanwhile, in regression, the algorithm derives output based on previous examples of input data, allowing for a backward analysis [68]. Unsupervised learning, on the other hand, is a form of ML where unlabeled input data is used in algorithms to produce patterns and relationships that provide new answers and questions that might have not been previously apparent to the investigators. There are two main subsets of unsupervised learning: clustering and dimensionality reduction. In clustering, algorithms specify and identify different patterns and establish different identification subgroups based on the heterogeneous input data given. Meanwhile, in dimensionality reduction, algorithms aim to decrease the number of subgroups identified with the input data given to simplify analysis and thereby draw fewer differences between different data [69].

Applications of Machine Learning in Pancreatic Cancer Detection

Using ML algorithms, we can apply different datasets that are relevant to pancreatic cancer, including imaging, biomarkers, histology slides, and more, introducing them into a desired algorithm. This could either be under supervised learning, like support vector machine, linear regression, and Naive Bayes, or under an unsupervised algorithm that generates output data like cluster analysis or automated ML. Thereupon, the algorithm runs through a great amount of information that could help researchers with the tedious manual labor of having to go through medical imaging or histology slides one at a time. Automating data analysis can help in diagnosing pancreatic cancer early on, especially with the help of large databases. ML could also potentially play a part in enhancing prognosis and treatment management in a more individualized approach [70].

Challenges and Opportunities in Implementing Machine Learning for Pancreatic Cancer Detection

One of the major obstacles to developing ML models for pancreatic cancer is the limited amount of good datasets with extensive and varied information on pancreatic cancer patients [71]. Another problem that ML models face is the imbalance in positive and negative cases potentially leading to biased predictions that can skew the results due to oversampling, undersampling, or even synthetic data generation. ML also often lacks interpretability, which would cause problems in healthcare systems adopting them due to low clinician trust. With the use of ML algorithms, datasets may be analyzed and small patterns suggestive of pancreatic cancer in its early stages might be found. To customize treatment regimens based on the unique features of each patient, ML can evaluate heterogeneous data sources, such as genetic information, tumor biomarkers, and patient demographics. By reducing side effects and optimizing therapy efficacy, precision medicine techniques powered by ML algorithms may lead to more focused and effective treatments [72].

Performance evaluation and validation

Metrics for Assessing Machine Learning in Pancreatic Cancer Studies

To arrange and illustrate the performance of ML classifiers, receiver operating characteristics (ROC) curves are used. The ROC curve is a line graph where the horizontal coordinate represents specificity and the vertical coordinate represents sensitivity. The evaluation metric is the area under the receiver operating characteristics curve (AUROC), and the higher the AUC value, the more effective the associated method is [73]. Aside from sensitivity and specificity, the other metrics frequently used to assess ML results are F1-Score, positive predictive value (PPV), and negative predictive value (NPV) [74]. Prediction ability is assessed using the relative risk (RR) and the AUROC curve. In earlier research, ML techniques were used in actual clinical records to estimate the risk of pancreatic cancer [75-78].

Navigating Data Limitations for Improved Patient Diversity

Because minorities are frequently underrepresented in various fields, AI suggests that insufficient data on minorities may make it difficult to evaluate patient diversity appropriately for algorithm development and testing. In addition, according to quality assessments of pancreatic imaging datasets, biliary stents or other reasons have rendered a significant fraction of CT pictures unsuitable for AI. Therefore, the quality of available images may hinder the construction of accurate AI models [79]. While radiomics, a technique that uses data characterization algorithms to extract specific elements from medical images, can aid in cancer diagnosis, limitations exist in terms of feature design, parameter setup, implementation, intra-individual test-retest repeatability, image-acquisition technology, multi-machine reproducibility, and segmentation reproducibility. The repeatability of radiomics is largely contested [80-82].

Real-World Application and Validation Studies

In 2022, Sandbank et al. introduced an AI system designed for breast biopsies, validated through a multi-site clinical trial, demonstrating high efficacy in identifying both in situ and invasive breast cancers. The AI algorithm, utilized as a second read application in pathology laboratories, displayed high accuracy, with a specificity of 98.27% and sensitivity of 98.51% for invasive carcinoma identification. External validation, involving 841 slides from two sites, confirmed the algorithm's robust performance across various subtypes. Implemented in Maccabi Healthcare Services, the AI system's real-time quality control effectively flagged suspicious cases, thereby reducing misdiagnosis rates. Despite some inconsistencies during deployment and external validation, the AI system notably improved diagnostic accuracy and clinical workflow efficiency [83].

Ethical considerations

In the evolving field of healthcare AI, ensuring ethical practices becomes paramount, particularly in addressing challenges related to confidentiality, privacy, data security, accountability, transparency, informed consent, and biases [84-86]. 

Confidentiality, Privacy, and Data Security

To create an efficient AI system, substantial amounts of data must be input to develop an accurate algorithm. This involves gathering sensitive information from various patient groups and physicians [85]. Delicate ethical considerations are required to strike a balance between accessing data in the name of public interest and upholding the right to privacy of an individual [85,87]. Moreover, since AI is essentially a computer system with data stored in drives, the risk of data loss or theft due to hacking is a real concern. Therefore, cybersecurity ultimately emerges as another area of focus concerning AI algorithms [88,89]. Hackers can access confidential patient data and interfere with it without necessarily tampering with the AI system itself. This not only breaches confidentiality but can also result in serious harm to patients [88].

Accountability

Navigating accountability-related aspects for mistakes presents an additional hurdle with the introduction of AI in medicine [90]. Because harm caused by AI is no longer linked to any individual decisions made by the physician, it is perceived as an inherent risk associated with the integration of AI into medicine [88]. Clear rules and laws are in place to handle situations where an error is made by an individual physician’s decision. However, attributing blame to a computer program may pose challenges when errors occur due to AI [90]. It is imperative to have policies and frameworks in place to clarify responsibility and accountability when medical errors occur [85].

Transparency and Informed Consent

Transparency is crucial for maintaining trust and when obtaining informed consent [84,87]. To make informed decisions, patients must have comprehensive information regarding their ailments, available diagnostic and treatment options, and the potential outcomes associated with these choices. With the introduction of AI, achieving such understanding necessitates a thorough grasp of AI tools and the way they contribute to the decision-making process by physicians who relay this information to patients [84]. A distinct ethical issue emerges when either the physician or the patient struggles to comprehend or interpret this information, thereby impeding the ability to make informed decisions [85].

Biases

Biases can also occur when programming AI. Although AI systems are trained using large amounts of data, biases based on demographics such as race or gender could creep in if the training data is not varied enough [88,91]. They may also stem from underlying assumptions by the creators of the algorithm or datasets [91]. If the data used while creating the algorithm is biased, the results will often reflect the same bias, leading to medical errors [85,90].

## Conclusions

The integration of AI in pancreatic cancer detection represents a significant advancement in oncology. With its roots dating back to the term's coinage in 1950 and its subsequent evolution alongside ML and DL, AI has emerged as a powerful tool in medicine. AI offers innovative solutions to improve patient outcomes, particularly in the realm of pancreatic cancer, where early detection is critical due to its late-stage presentation and limited eligibility for curative surgical intervention. Current methods for pancreatic cancer detection, including histopathological assessment, tumor markers, clinical findings, and molecular imaging, have shown promise but have certain limitations. AI-driven approaches have the potential to overcome these challenges by analyzing complex datasets, including imaging data, biomarker profiles, and clinical parameters, to enhance early detection and prognosis prediction. 

Moreover, AI-enabled imaging technologies, such as DLAD algorithms, have demonstrated the ability to augment traditional imaging methods, thereby improving both accuracy and efficiency. However, the implementation of AI in healthcare requires careful consideration of various ethical concerns, including patient privacy, data security, accountability, transparency, and biases. Despite these challenges, the integration of AI holds great promise for advancing personalized medicine in pancreatic cancer and other complex diseases. By addressing ethical considerations and leveraging AI's capabilities, healthcare systems can enhance early diagnosis, tailor treatment strategies, and ultimately improve patient outcomes. Continued research, collaboration, and ethical stewardship are essential to harnessing the full potential of AI in oncology and realizing its transformative impact on patient care.
